# Editorial: Methodological approaches for fish reproduction management

**DOI:** 10.3389/fphys.2023.1271809

**Published:** 2023-10-04

**Authors:** Alejandro S. Mechaly, Jorge M. O. Fernandes, Benjamín Costas, Elvira Fatsini

**Affiliations:** ^1^ Instituto de Investigaciones en Biodiversidad y Biotecnología (INBIOTEC-CONICET), Mar del Plata, Argentina; ^2^ Fundación para Investigaciones Biológicas Aplicadas (FIBA), Mar del Plata, Argentina; ^3^ Faculty of Biosciences and Aquaculture, Nord University, Nord, Norway; ^4^ CIIMAR—Centro Interdisciplinar de Investigação Marinha e Ambiental, Matosinhos, Portugal; ^5^ Abel Salazar Institute of Biomedical Sciences (ICBAS), University of Porto, Porto, Portugal; ^6^ Center of Marine Sciences-CCMAR, University of Algarve, Faro, Portugal

**Keywords:** fish, reproduction, animal welfare, aquaculture, omics

Until recently, molecular and genomic knowledge of fish reproduction focused mainly on a few model species relevant to fundamental biology and human biomedical research, such as zebrafish (*Danio rerio*) and medaka (*Oryzias latipes*). Studying the complex reproductive processes in fish is a major challenge, as there are nearly 35,000 fish species, all with their own reproductive strategies and systems. In the last two decades, the use of cultured fish has increased significantly for both basic research and aquaculture. This is mainly due to the fact that fish farming is the fastest-growing sector of animal food production worldwide (FAO, 2020). Therefore, the growing interest in diversifying markets, adopting sustainable aquaculture production practices, consolidating farmed species, and implementing biodiversity conservation strategies has led to the development of new commercial fish models.

The study of fish reproduction in aquaculture is essential for improving farming practices and ensuring sustainable production (Mechaly et al., 2023). Animal welfare is becoming increasingly important in both laboratory research and aquaculture, and the ethical treatment of laboratory animals requires special committees to monitor research protocols involving fish (Toni et al., 2019). Fish welfare is an important and neglected aspect of animal care that encompasses the wellbeing and ethical treatment of fish species (Grunow and Strauch, 2023). Over the past decade, interest has increased in developing non-lethal and less invasive methods to study fish reproduction.

This Research Topic has been explored in six articles addressing various aspects of fish reproduction, and using state-of-the-art methods such as transcriptomics and transgenesis.

The first article examined the effects of recombinant gonadotropin hormones (rGths) on vitellogenesis in the flathead grey mullet (*Mugil cephalus*). Ovary samples were collected at four different developmental stages, and RNA-seq analysis was performed to identify differentially expressed genes. As vitellogenesis progressed, more genes were upregulated than downregulated. Application of recombinant follicle-stimulating hormone (rFsh) induced ovarian development from previtellogenesis to early mid-vitellogenesis, enriching signaling pathways related to ovarian steroidogenesis, lipid metabolism, and cell-cell adhesion. Combined application of rFsh and recombinant luteinizing hormone (rLh) promoted oocyte growth to late vitellogenesis, involving signaling pathways related to energy production, and rLh application induced completion of vitellogenesis and oocyte maturation. These results provide valuable insights for the use of rGths in aquaculture and in the conservation of endangered species (Ramos-Júdez et al.).

A second study focused on the Peruvian grunt (*Anisotremus scapularis*); a commercial fish species important for aquaculture development in Peru. Seasonal samplings of wild catches from 2 years were used to determine the female reproductive cycle and reproductive strategy, which was classified as asynchronous spawning with indeterminate fertility. The average size at first sexual maturity (L100) was determined to be 25.3 cm and the spawning period was determined to be from late spring to mid-autumn. A thermophotoperiodic breeding program based on environmental parameters was also proposed. These results provide important insights for improving the aquaculture of *A. scapularis* (Carrera Santos et al.).

Another study compared the effects of two hormones, 17α,20β-dihydroxy-4-pregnen-3-one (DHP) and progesterone (P), on ovulation and egg quality in European eel (*Anguilla anguilla*). In the *in vitro* experiment, both DHP and P induced oocyte maturation at specific doses. Gene expression related to maturation and ovulation was similar in both treatments. In the *in vivo* experiment, females injected with either DHP or P showed no significant differences in ovulation, egg release, or larval viability. RNA sequencing showed similar effects on genes related to egg quality with both hormones. Considering the significant cost difference, this study suggests that P is a more cost-effective alternative to DHP for inducing ovulation in European eels (Jehannet et al.).

Two other articles focused on basic research aimed at understanding the molecular mechanisms of gonadotropin-inhibitory hormone (GnIH) action on fish reproduction. One of these studies investigated the interactions between GnIH and neuropeptide FF receptor 2 (NPFFR2) in the European sea bass (*Dicentrarchus labrax*). It was found that GnIH peptides can activate NPFFR2-1 and NPFFR2-2, resulting in the reduction of forskolin-induced CRE-luc activity. On the other hand, NPFF and NPAF stimulated SRE-luc activity via NPFFR2-1 and NPFFR2-2. GnIH2 inhibited NFAT-RE-luc activity in COS-7 cells expressing NPFFR2-1. However, neither GnIH nor NPFF peptides affected ERK phosphorylation levels via NPFFR2 receptors. These findings suggest that sea bass GnIH peptides may partially exert their functions through NPFFR2, potentially involving PKA, PKC, and Ca^2+^ signaling pathways (Wang et al.). The other study explored the regulatory role of GnIH in neurosteroid synthesis and its impact on behaviour in male sea bass. Intracerebroventricular injection of sbGnIH2 decreased transcript levels of *3b-hsd* and *17b-hsd*, while increasing *cyp19b* expression (brain aromatase). GnIH- and aromatase-positive cell interactions suggest paracrine and neuroendocrine actions mediating GnIH effects on aromatase. Pituitary expression of *17b-hsd* and estrogen receptors was also reduced. The mirror test showed sbGnIH-2’s influence on aggressive behaviour, with decreased interaction with the mirror and altered mirror zone activities, while locomotor activity remained unaffected. These findings reveal GnIH’s role in regulating neurosteroid-synthesizing enzymes and aggressive behaviour in sea bass (Paullada-Salmerón et al.).

The last article focused on the development of genetic tools that could be useful to control fish reproduction and to study housekeeping genes involved in development and mass gains. In this study, the authors discuss the challenges and limitations encountered in using Cre/Lox technology in zebrafish genetics and propose a new set of tools to improve efficiency and reliability. They introduced a codon-improved Cre version (iCre) to address issues like silencing, mosaicism, and partial recombination. Additionally, they created tol2-kit compatible vectors for easy generation of iCre-mRNA and iCre-transgenes for transient and transgenic experiments. Interestingly, they found that maternal iCre-mRNA or protein deposition from female transgenics resulted in complete Lox-responder transgene conversion, unlike using male drivers or mRNA injections. They also explore the use of exogenous CRE-protein for robust and homogeneous Lox-recombination. Overall, these tools offer promising applications for zebrafish genetics research, especially for investigating difficult-to-manipulate genes involved in development, sex determination, and reproduction (Tromp et al.).

The Research Topic editors of this Research Topic strongly advocate the use of non-lethal methods in addition to existing technical approaches that traditionally sacrifice fish. The inclusion of non-lethal and less invasive methods not only improves our understanding of fish reproduction but also addresses the increasing concerns of animal welfare and ethical research practices. We also thank the authors and the reviewers for their excellent results and methods for studying fish reproduction presented here, which greatly contribute to our current understanding of fish reproduction and may be applied to improve the sustainability of the aquaculture sector.
